# Sphingolipids in Ventilator Induced Lung Injury: Role of Sphingosine-1-Phosphate Lyase

**DOI:** 10.3390/ijms19010114

**Published:** 2018-01-01

**Authors:** Vidyani Suryadevara, Panfeng Fu, David Lenin Ebenezer, Evgeny Berdyshev, Irina A. Bronova, Long Shuang Huang, Anantha Harijith, Viswanathan Natarajan

**Affiliations:** 1 Department of Bioengineering, University of Illinois at Chicago (UIC), Chicago, IL 60607, USA; vsurya2@uic.edu; 2 Department of Pharmacology, University of Illinois at Chicago (UIC), Chicago, IL 60612, USA; pfu@uic.edu (P.F.); debene2@uic.edu (D.L.E.); lhuang82@uic.edu (L.S.H.); 3 Department of Pharmacology, Department of Medicine, National Jewish Health Center, Denver, CO 80206, USA; BerdyshevE@njhealth.org (E.B.); bronovai@njhealth.org (I.A.B.); 4 Department of Pediatrics, University of Illinois at Chicago (UIC), Chicago, IL 60612, USA; harijith@uic.edu; 5 Department of Medicine, University of Illinois at Chicago (UIC), Chicago, IL 60612, USA

**Keywords:** sphingolipids, sphingosine-1-phosphate, S1P lyase, sphingosine kinases, lung injury, mechanical ventilation

## Abstract

Mechanical ventilation (MV) performed in respiratory failure patients to maintain lung function leads to ventilator-induced lung injury (VILI). This study investigates the role of sphingolipids and sphingolipid metabolizing enzymes in VILI using a rodent model of VILI and alveolar epithelial cells subjected to cyclic stretch (CS). MV (0 PEEP (Positive End Expiratory Pressure), 30 mL/kg, 4 h) in mice enhanced sphingosine-1-phosphate lyase (S1PL) expression, and ceramide levels, and decreased S1P levels in lung tissue, thereby leading to lung inflammation, injury and apoptosis. Accumulation of S1P in cells is a balance between its synthesis catalyzed by sphingosine kinase (SphK) 1 and 2 and catabolism mediated by S1P phosphatases and S1PL. Thus, the role of S1PL and SphK1 in VILI was investigated using *Sgpl1^+/−^* and *Sphk1^−/−^* mice. Partial genetic deletion of *Sgpl1* protected mice against VILI, whereas deletion of SphK1 accentuated VILI in mice. Alveolar epithelial MLE-12 cells subjected to pathophysiological 18% cyclic stretch (CS) exhibited increased S1PL protein expression and dysregulation of sphingoid bases levels as compared to physiological 5% CS. Pre-treatment of MLE-12 cells with S1PL inhibitor, 4-deoxypyridoxine, attenuated 18% CS-induced barrier dysfunction, minimized cell apoptosis and cytokine secretion. These results suggest that inhibition of S1PL that increases S1P levels may offer protection against VILI.

## 1. Introduction

Acute lung injury (ALI) is a cause of acute respiratory failure in patients experiencing sepsis, pneumonia, gastric aspiration, and trauma [1]. Patients with ALI develop a protein-rich pulmonary edema resulting from exudation of fluid into the interstitial space of the lung. The pathobiological basis of these changes results in increased permeability of the vascular barrier, a hallmark of ALI. One pathophysiological consequence of pulmonary edema is accumulation of fluid in the lung interstitial spaces that results in impaired gas exchange [2], one of the reasons why assisted ventilation is required to support most patients. However, extended duration of mechanical ventilation (MV) is associated with ventilator-induced lung injury (VILI), which is a highly morbid clinical entity caused by excessive mechanical stress in the lung [3]. VILI is characterized by significant structural changes in the lungs, wherein there is a loss of alveolar permeability, influx of pro-inflammatory cytokines and alveolar epithelial apoptosis [4]. There is also increased pulmonary pressure, reduced compliance and increased physiological dead space in the lungs.

Though pressure and volume settings of the ventilator play a major role in VILI, the cellular mechano-transduction events also play a crucial role in mediating VILI. There is mechano-transduction of the physical forces due to mechanical stretch of pulmonary endothelial and alveolar epithelial cells that lead to biochemical changes at the cellular level. This cellular mechano-transduction leads to stress fiber reorientation, remodeling of the extracellular matrix (ECM) components in the cell, cell-cell adhesions as well as cell-ECM adhesion, activation of several nuclear transcription factors, and secretion of inflammatory cytokines. Several recent studies have addressed signaling mechanisms that contribute to VILI [5]. Mechanical forces arising from MV has been shown to directly stretch cell membrane, and rapidly induce phosphorylation and activation of receptors [6], cation channels [7], phospholipases [8], and signaling pathways such as protein kinase C, mitogen-activated protein kinases, and transcriptional factors that regulate lung inflammation and injury [9,10,11].

Phospholipids and sphingolipids are membrane lipids that are ubiquitously present in all eukaryotic cells. In addition to being the structural components of cell membranes, recent studies have shown that both phospholipids and sphingolipids and their metabolites serve as intracellular and extracellular signaling molecules in normal and several human pathologies [12]. The predominant sphingolipid in the cell is sphingomyelin (SM) and its hydrolysis by sphingomyelinase leads to production of ceramide [13]. Ceramide is an important lipid mediator that regulates growth arrest and apoptosis [14], and is deacylated by ceramidases to sphingosine [15]. Further, in cells, ceramidase converts ceramide to sphingosine, which is phosphorylated by sphingosine kinase (SphKs) 1 and 2 to sphingosine-1-phosphate (S1P).

S1P is a pleotropic bioactive sphingolipid involved in several cellular functions and signals both intracellularly and extracellularly via G-protein coupled S1P_1-5_ receptors on the plasma membrane of cells [16]. S1P levels in cells are regulated by its synthesis and degradation. S1P is converted back to sphingosine by S1P phosphatases (SPPs) and irreversibly degraded by a pyridoxal dependent enzyme, S1P lyase (S1PL) to ∆2-hexadecenal and phosphoethanolamine [17]. Recent studies suggest a key role for S1P signaling in several lung pathologies including sepsis [18], pulmonary artery hypertension [19], pulmonary fibrosis [20,21,22,23], asthma [24] and bronchopulmonary dysplasia [25]. A potential role for dysregulated sphingolipid metabolism in VILI is intriguing in consideration of S1P signaling contributions to several other lung pathologies [26,27].

Previous studies have shown that ceramide levels were elevated in tracheal aspirates of patients subjected to conventional mechanical ventilation [28]. Further, animal models with the appropriate ventilator settings have been used as the preclinical model to study the effects of mechanical ventilation [29]. It was shown that serum of rats subjected to mechanical ventilation had increased dihydrosphingosine (a precursor of ceramide) levels, whereas reduced sphingosine levels [30]. These changes in the sphingolipid metabolites in the serum showed a significant correlation with the peak inspiratory pressure (PIP) and lung injury score. However, there are few reports on the role of sphingolipids and sphingoid bases in VILI [28,30,31], and this study we investigated the effect of MV on sphingoid bases levels and sphingolipid metabolizing enzymes in the pathogenesis of murine VILI.

## 2. Results

### 2.1. Mechanical Ventilation Modulates Sphingoid Bases Levels and Expression of S1P Lyase in Mouse Lung

**Figure 1:**
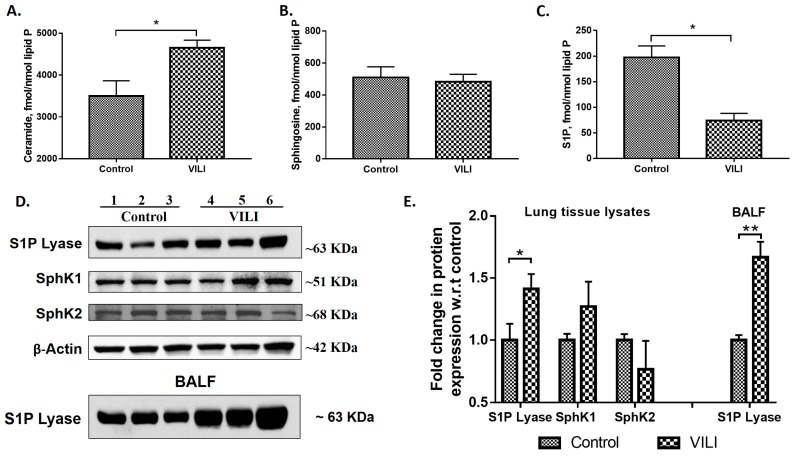
Ventilator-induced lung injury (VILI) modulates sphingolipid metabolism in mouse lung: C57BL/6J mice were mechanically ventilated at 0 PEEP (Positive End Expiratory Pressure), 30 mL/kg for 4 h, lungs were lavaged, lipids from lung tissues were extracted and analyzed by LC-MS/MS to quantify ceramides (**A**), sphingosine (**B**), and sphingosine-1-phosphate (S1P) (**C**). Values are means ± SEM of three independent experiments. Total lung tissue lysates from control and mechanically ventilated mice from above were subjected to Western blotting (**D**). Shown is a representative Western blot of S1PL expression in lung tissue and bronchoalveolar lavage fluid (BALF), and sphingosine kinase (SphK) 1 & 2 and β-actin in lung tissues. (**E**), Quantification of the western blots as seen in (**D**) by densitometry and fold changes were normalized to β-actin in lung tissue lysates. * *p* < 0.05, ** *p* < 0.005 (*n* = 3 to 5 per group).

MV of mouse lungs resulted in a significant increase in ceramide (~1.5-fold) (Figure 1A) and a decrease in S1P (~2.5 fold) (Figure 1B) levels as compared to animals with spontaneous breathing; however, there was no change in sphingosine level (Figure 1C) between the two groups. As S1P levels in tissues are regulated by its synthesis and catabolism, the effect of MV on expression of SphK1 & 2 and S1PL was determined. MV of mouse lungs for 4 h enhanced S1PL expression approximately 1.5-fold in the lung tissue; however, did not affect the expression of SphK1 or SphK2 as determined by immunoblotting (Figure 1D,E). In addition to the lung tissue, MV increased S1PL expression in bronchoalveolar lavage fluid (BALF) (Figure 1D). These results suggested that MV differently altered sphingoid bases levels and reduction in S1P levels correlated with the increased S1PL protein expression in mouse lungs.

### 2.2. Genetic Deletion of S1PL, but Not Sphk1, Attenuates Ventilator-Induced Lung Injury and Inflammation

**Figure 2:**
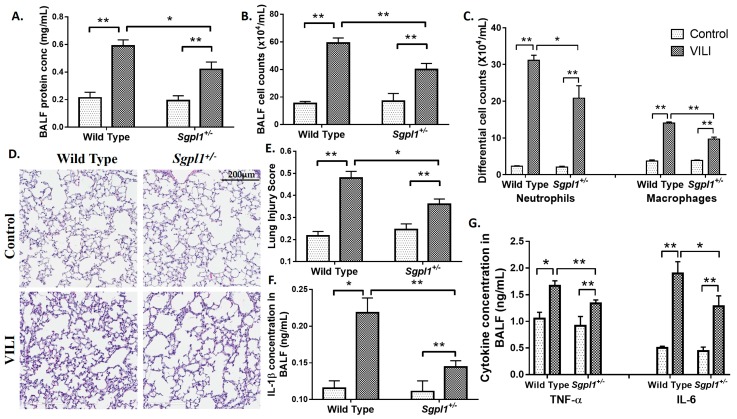
Partial deletion of S1P Lyase (*Sgpl1^+/−^*) in mice confers protection against mechanical ventilation-mediated lung injury: 129/SV and *Sgpl1^+/−^* mice were subjected to mechanical ventilation for 4 h at 0 PEEP, 30 mL/kg tidal volume. Lungs were lavaged and bronchoalveolar lavage (BAL) fluids were analyzed for (**A**) total protein, (**B**) total cell counts, and (**C**) differential cell counts. (**D**) lung tissue sections from *Sgpl1^+/+^* and *Sgpl1^+/−^* mice with or without mechanical ventilation, stored in formalin were processed for staining with hematoxylin & eosin (H&E) for infiltration of cells into alveolar space. Shown is a representative staining of the lung tissue from three independent experiments, and lung injury score was quantified (**E**). BAL fluids were also analyzed for secretion of IL-β (**F**), IL-6 and TNF-α (**G**) using enzyme-linked immunosorbent assay (ELISA) kits. * *p* < 0.05 and ** *p* < 0.005 (*n* = 3 to 5 per group). Scale bar = 200 μm.

To assess the role of S1PL in VILI, wild type (WT) and *Sgpl1^+/−^* (heterozygous) mice were subjected to MV as deletion of both alleles of *Sgpl1* gene in mice results in defective vascular development and lethal after 4 weeks of birth [32]. As determined earlier, S1PL mRNA and protein expression in *Sgpl1^+/−^* mice were reduced approximately 50% compared to WT mice and S1P levels in plasma and lung tissues were significantly higher compared to the WT animals [33]. After MV for 4 h, lungs were lavaged and BAL fluids were collected and analyzed. The BAL total protein concentration, a measure of capillary permeability, was significantly elevated (~3 fold) in MV-challenged WT mice as compared to non-ventilated animals (Figure 2A). BAL fluid protein concentration was significantly decreased in *Sgpl1^+/−^* mice treated with MV as compared to WT mice exposed to MV (Figure 2A). Evaluation of BAL fluid from WT mice after 4 h MV revealed significant increase in total cell counts (Figure 2B), which was predominantly neutrophils and macrophages (Figure 2C). Knockdown of one allele of *Sgpl1* gene decreased infiltration of both neutrophils and macrophages into the alveolar space after MV (Figure 2C). Compared with non-ventilated littermate control WT mice, MV induced inflammatory cell infiltration in lung interstitium and alveolar space (Figure 2D), and partial deletion of *Sgpl1^+/−^* significantly attenuated MV-induced lung injury (Figure 2D,E). Similarly, MV-induced inflammatory cytokine levels of IL-1β, TNF-α and IL-6 in BAL fluids were significantly reduced in *Sgpl1^+/−^* mice compared to WT counterparts (Figure 2F,G).

**Figure 3:**
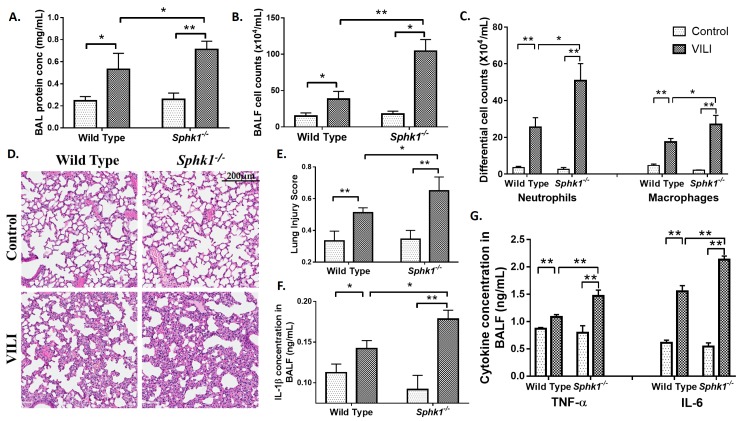
Genetic deletion of SphK1 accentuates mechanical ventilation-induced lung injury in mice. *Sphk1^+/+^* and *Sphk1^−/−^* mice (6–8 weeks) in C57BL/6J background were subjected to mechanical ventilation for 4 h at 0 PEEP, 30 mL/kg tidal volume. Lungs were lavaged and BAL fluids were analyzed for (**A**), total protein concentration (**B**), total cell counts (**C**), differential cell counts. (**D**), lung tissue sections from *Sphk1^+/+^* and *Sphk1^−/−^* mice with or without mechanical ventilation, stored in formalin were stained with hematoxylin & eosin for infiltration of cells into alveolar space. Shown is a representative staining of the lung tissue (**D**) and further lung injury score was quantified with histopathological observations from H&E staining of the sections from the lung tissue (**E**). BAL fluids were also analyzed for secretion of IL-β (**F**), IL-6 and TNF-α (**G**) using ELISA kits. * *p* < 0.05, and ** *p* < 0.005 (*n* = 3 to 5 per group). Scale bar = 200 μm.

**Figure 4:**
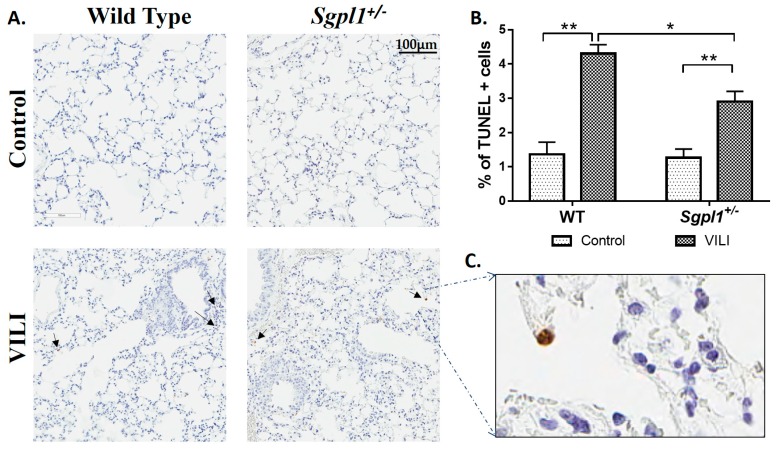
Partial deletion of S1P lyase (*Sgpl1^+/−^*) in mice confers protection against mechanical ventilation-mediated apoptosis: *Sgpl1^+/+^* and *Sgpl1^+/−^* mice in 129/SV background were subjected to mechanical ventilation for four hours at 0 PEEP, 30 mL/kg tidal volume. Lung tissue sections from *Sgpl1^+/+^* and *Sgpl1^+/−^* mice with or without mechanical ventilation, stored in formalin, were processed to study ventilation induced apoptosis by TUNEL assay. (**A**) Shown is a representative section from the TUNEL staining that indicates that there was more apoptosis in the lungs of WT mice compared to *Sgpl1^+/−^* subjected to ventilation, scale bar = 100 µm. (**B**) The average of number of TUNEL positive cells/total number of cells in each field. (**C**) Higher magnification at 40× indicated that the TUNEL-positive cells are mostly epithelial cells. * *p* < 0.05 and ** *p* < 0.005 (*n* = 3 to 5 per group).

In contrast to the *Sgpl1^+/−^* mice, *Sphk1^−/−^* mice had higher protein concentration (Figure 3A), higher total cell counts (Figure 3B), higher infiltration of alveolar neutrophils and macrophages (Figure 3C) in BAL fluids as compared to the WT mice. Also, *Sphk1^−/−^* mice were more prone to lung injury than WT control mice, as seen from the H&E staining (Figure 3D,E). Likewise, the cytokine levels in BAL fluids were higher in *Sphk1^−/−^* mice than in WT mice (Figure 3F,G). Further, the number of TUNEL (Terminal deoxynucleotidyl transferase-mediated dUTP nick-end labelling) positive cells undergoing apoptosis was reduced in *Sgpl1^+/−^* mice as compared to the WT counterpart subjected to MV (Figure 4). These results suggest an inflammatory/injury role for S1PL and a protective role for SphK1 in VILI.

### 2.3. Cyclic Stretch Modulates Sphingoid Bases Levels and S1P Lyase Expression in Lung Epithelial Cells

**Figure 5:**
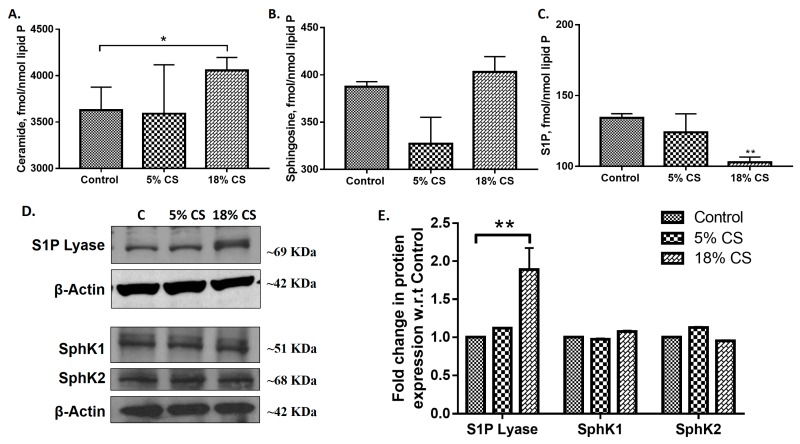
Cyclic stretch (CS) alters sphingoid bases levels in alveolar epithelial cells: Alveolar epithelial MLE-12 cells grown in BioFlex plates to ~90% confluence were subjected to 0% (static), 5% or 18% CS for 48 h, lipids were extracted and sphingoid bases levels were determined by LC-MS/MS to quantify ceramides (**A**), sphingosine (**B**), and sphingosine-1-phosphate (S1P) (**C**). Values are means ± SEM of three independent experiments. Total cell lysates from control and cyclic stretched MLE-12 cells from above were subjected to Western blotting (**D**). Shown is a representative Western blot of S1PL, SphK1, SphK2 and β-actin in total cell lysates from three independent experiments in triplicate. (**E**), Quantification of the Western blots from (**D**) by densitometry and fold changes were normalized to β-actin. * *p* < 0.05, ** *p* < 0.005.

Pathophysiological condition of mechanical stretch is represented by 18% linear elongation with equibiaxial stretch (0.2 Hz, 25 cycles/min, sinusoidal wave), whereas 5% mechanical stretch simulates physiological conditions. Having established that MV modulates sphingoid bases levels and S1PL expression in vivo, next we determined the consequence of pathological cyclic stretch (CS) on sphingolipids and its metabolizing enzymes in alveolar epithelial cells. Mouse alveolar MLE-12 cells were subjected to 5% and 18% CS for 48 h with a non-stretched plate as control. Analysis of sphingoid bases levels by LC-MS/MS in MLE-cells subjected to different magnitudes of CS revealed an increase in ceramide levels after 18% CS compared or 5% CS or no stretch while a significant decrease in S1P level was observed after 18% CS (Figure 5A,B). However, no change in sphingosine levels was seen after 18% CS of MLE-12 cells as compared to static or 5% CS. 18% CS elevated S1PL expression with no significant changes in SphK1 and SphK2 expression levels (Figure 5C,D). These results established that 18% CS modulated sphingoid bases levels and expression of S1PL in alveolar epithelial cells was like MV of mouse lungs.

### 2.4. Inhibition of S1P Lyase by 4-Deoxypyridoxine Attenuates Cyclic Stretch-Induced Epithelial Cell Apoptosis

**Figure 6:**
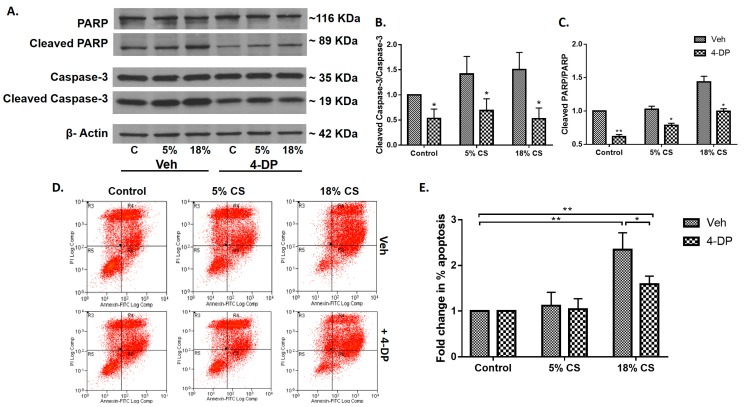
Inhibition of S1P Lyase attenuates cyclic stretch induced apoptosis in alveolar epithelial cells: Alveolar epithelial MLE-12 cells grown in BioFlex plates to ~90% confluence were subjected to static, 5% or 18% CS for 48-h in the absence or presence of S1PL inhibitor, 4-DP (1 mM), and cell lysates were subjected to Western blotting and immuno stained for Caspase 3, cleaved Caspase-3, PARP and cleaved PARP (**A**). Programmed cell death following 18% CS of MLE-12 cells was indicated by cleavage of Caspase-3 & PARP, indicators for apoptosis, which was reduced upon pretreating the cells with 4DP (**A**). The western blots from (**A**) were quantified for cleaved Caspase-3 (**B**), and PARP (**C**) by densitometry analysis. Values are means ± SD from three independent experiments. The role of S1P lyase activity in CS-induced apoptosis was further confirmed by flow cytometry analysis to sort early apoptotic and late apoptotic cells. (**D**), Apoptotic cells were identified by double labelling with Annexin V and PI or by labelling with only annexin V. PI labels all dead cells, including necrotic cells and cells in late stages of apoptosis; cells entering early apoptosis are stained only by annexin V, and the viable cells do not stain with annexin V or PI. (**D**) shows representative dot plots of annexin V and PI staining. The region R5 indicates viable cells (V-FITC−/PI−); the region R6, cells in early apoptosis (V-FITC+/PI−); the region R4, cells in late apoptosis (V-FITC+/PI+); and the region R3, necrotic cells (FITC−/PI+). The late apoptotic cells stained as Annexin V+/PI+ cells seen on R4, and early apoptotic cells were stained as Annexin V+/PI− cells seen on R6. (**E**), the percentage of late apoptotic cells after 0%, 5% and 18% cyclic stretch were quantified. * *p* < 0.05 and ** *p* < 0.005.

Having demonstrated that *Spgl1^+/−^* mice were protected from VILI, next we investigated the role of S1PL activity in CS-induced epithelial cell apoptosis. Inhibition of S1PL was achieved by pre-incubating cells with 4-deoxypyridoxine (4-DP), an analog of pyridoxal phosphate [33]. We have shown earlier that 4-DP effectively reduced S1PL activity as determined by elevated intracellular and secreted S1P levels in human lung endothelial cells [33,34]. Alveolar MLE-12 epithelial cells were pretreated with 4-DP (1 mM) for 1 h prior to 48 h mechanical CS. Both 5% and 18% CS increased cleaved Poly (ADP-ribose) polymerase (PARP) and caspase-3 compared to non-stretched MLE-12 cells, which was blocked by 4-DP (Figure 6A–C). Additionally, CS-induced apoptosis was confirmed by flow cytometry analysis of the cells that were double labelled with Annexin V and Propidium Iodide (PI). There was a significant increase in late apoptotic cells in 18% CS as indicated by Annexin V-FITC+ as well as PI+ cells (Figure 6D,E). Furthermore, suppression of apoptosis was observed in cells that were pre-treated with 4-deoxypyridoxine (Figure 6D,E). These results showed a role for S1PL in CS mediated apoptosis in epithelium.

### 2.5. Inhibition of S1P Lyase Reduces Cyclic Stretch Induced Paracellular Gap Formation and Cytokine Secretion

**Figure 7:**
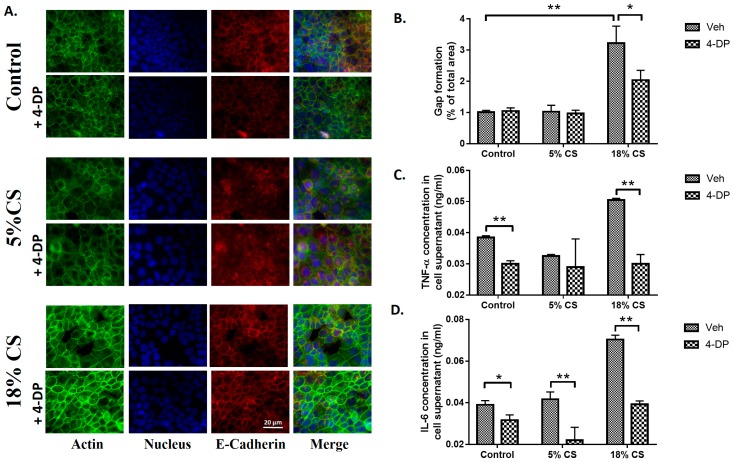
Inhibition of S1P Lyase attenuates cyclic stretch-induced paracellular gap formation and cytokine secretion: Alveolar epithelial MLE-12 cells grown in BioFlex plates to ~90% confluence were subjected to static, 5% or 18% cyclic stretch (CS) in the absence or presence of S1PL inhibitor, 4-deoxypyridoxine (4-DP) (1 mM) for 48 h. Inhibition of S1PL with 4-DP reduced CS induced paracellular gap formation (**A**). Shown is a representative photomicrograph of the MLE-12 cells stained with phalloidin green for actin (green), E-Cadherin (red) and nucleus marker DAPI (Blue). Images taken under 20× magnification. (**B**) Quantification of the gap formation in (**A**) by percentage of gaps per total area. The supernatant from (**A**) were analyzed for inflammatory cytokines TNF-α (**C**) and IL-6 (**D**) levels by ELISA. Values are mean ± SD from three independent experiments. * *p* < 0.05 and ** *p* < 0.005. Scale bar = 20 μm.

Having demonstrated that partial deletion of *Sgpl1 gene* (*Sgpl^+/−^*) in mice attenuated VILI and epithelial apoptosis, next we determined the role of S1PL in CS-induced para-cellular gap formation and secretion of pro-inflammatory cytokines in alveolar epithelial cells. Treatment of MLE-12 cells with 4-DP (1 mM) showed a dramatic reduction in pathological 18% CS-induced paracellular gap formation that was visualized by staining for actin and quantification of merged (yellow) of actin (green) and E-cadherin (red) in alveolar MLE-12 cells (Figure 7A,B). Furthermore, in MLE-12 cells, 4-DP blocked CS-induced secretion of IL-6 and TNF-α induced by CS (Figure 7C,D). These results showed a role for S1PL in epithelial barrier function and secretion of pro-inflammatory cytokines in CS-induced VILI.

## 3. Discussion

Mechanical ventilation (MV) is done in patients with injured lungs as in the case of acute respiratory distress syndrome, to improve the lung function. MV has been shown to lead to over distention of the alveolar epithelium and alveolar epithelial apoptosis [35]. Ventilation stimulates recruitment of inflammatory cells in the lung and the subsequent development of systemic inflammation [36] resulting in damage to the alveolar epithelium, increase in epithelial and endothelial permeability in the lung, apoptosis, reorganization of the stress fibers and extracellular matrix deposition at the cellular level [37]. The current study demonstrates a critical role of sphingolipids and sphingolipid metabolizing enzyme(s) in regulating VILI.

Sphingolipids and sphingolipid metabolizing enzymes differentially modulate various respiratory diseases [16,26,27]. Previous studies have identified alterations in sphingolipid metabolites like ceramides and dihydrosphingosine during mechanical ventilation in patients and rats respectively [28,30]. Ceramides are shown to play an important role in apoptosis by releasing soluble factors, which have been found to mediate inflammation through the JNK pathway [38] and contribute to caspase-1 activation which is dependent on Nlrp3 inflammasome [39]. Ceramide also plays a vital role in inflammation as they mediate cytokine release and neutrophil sequestration during acute lung injury [40].

Ceramide levels have been shown to be increased in patients subjected to conventional MV [28]. We speculate that despite increases in ceramide levels associated with lung protective ventilation strategies in neonates, the beneficial effects of ventilating at low tidal volumes due to decreased mechanical lung stress likely outweigh any injurious effects of increased ceramide [41]. Separately, we relied on a well characterized and widely used murine VILI model. Nonetheless, one limitation of our study is the use of anesthesia in both controls (spontaneously breathing) and VILI-challenged mice given the potential for side effects on respiratory and hemodynamics associated with the administration of anesthetics. Direct comparison would require either protectively ventilated animals or animals sacrificed soon after sedation and intervention. However, our results, firmly support effects on ceramide levels specific to MV that were not observed in spontaneously breathing animals, consistent with the literature describing similar effects in neonates subject to MV.

We found that ceramide levels to be elevated in the lung after MV and in alveolar epithelial cells subjected to cyclic stretch (Figure 1 and Figure 5). Increased ceramide levels have been attributed to epithelial cell apoptosis in cystic fibrosis (CF) lung tissues [42] and endothelial apoptosis due to chronic exposure to cigarette smoke [43]. In VILI, a decrease in S1P level in lung tissue or epithelial cells could also contribute to apoptosis as S1P is anti-apoptotic in many cell types. In this study, we demonstrate the importance of S1P signaling in VILI and assessed the role of S1P-mediated cellular responses are regulated by its synthesis, catalyzed by SphKs [44], and degradation mediated by SPPs [45] and S1PL [46].

The data presented in this study show for the first time that MV of mice for 4 h resulted in a significant decrease in S1P levels in lung tissues compared to non-mechanical ventilated mouse lungs. This decrease in S1P in the lung tissue directly correlated with enhanced expression of S1PL, and partial genetic deletion of *Sgpl1* in mice conferred protection against VILI by attenuating alveolar epithelial cell apoptosis in vivo*.* Also, the decrease in S1P levels after MV of animals or cyclic stretch of MLE-12 cells could be due to changes in ceramidase expression or activity. In cells, ceramidase hydrolyses ceramide to sphingosine, which is subsequently converted to S1P by SphKs 1 & 2. In the present study, we did not determine the expression or activity of ceramidase in MV mouse lungs or cyclic stretched epithelial cells. We further demonstrated that MV-mediated secretion of IL-1β, IL-6 and TNF-α and protein leak in the alveolar space was attenuated in *Sgpl1^+/−^* mice compared to WT controls. However, genetic deletion of SphK1 in mice exacerbated MV-induced lung inflammation and injury suggesting a protective role for SphK1 and S1P signaling in VILI. There are two possibilities for accentuation of VILI in SphK1 KO mice: (1) Although SphK1 and SphK2 expressions are not altered in VILI, their activity could be modulated (inhibited) by oxidative stress, which might contribute to a decrease in S1P levels. S1P levels are significantly lower (~30% compared to WT mice) and since S1P levels play a critical role in VILI; (2) Mechanical ventilation or cyclic stretch increased the expression of S1P lyase in mouse lungs or MLE-12 cells, which could alter S1P levels in mouse lungs accounting for accentuated lung injury both in vivo and in vitro. Thus, our findings provide new insights into potential mechanism(s) of intracellularly accumulating S1P in ameliorating MV-induced lung injury and inflammation provide a novel therapeutic strategy to limit MV-associated morbidity and mortality.

Genetic or siRNA deletion of SphK1 has been shown to augment LPS-induced lung injury most likely due to less S1P production in the lung [34]. Similar to the LPS model [34], in the present study we observed that MV-induced VILI was accentuated in *Sphk1^−/−^* mice; however, the involvement of SphKs in lung inflammatory injury depends on the lung pathology. *Sphk1^−/−^* mice exhibited enhanced pulmonary leak and injury to LPS while adenoviral delivery of SphK1 gene to *Sphk1^−/−^* mice decreased LPS-mediated vascular leak and inflammation suggesting a protective role for SphK1 [34]. However, reduction of SphK1 expression by genetic deletion or siRNA or inhibition of activity decreased allergen-induced airway inflammation in ovalbumin sensitized and challenged murine model of asthma [47]. In contrast to this finding, airway hyper-responsiveness was reduced in the absence of SphK1 in an acute allergen challenged model of inflammation in mice [48]. Knockdown of SphK1 in mice conferred protection against hypoxia-induced pulmonary artery hypertension [49], hyperoxia-induced lung injury in neonatal bronchopulmonary dysplasia [25] and bleomycin-induced pulmonary fibrosis [22] but not in VILI as demonstrated here. Thus, differential modulation of S1P levels has a disparate role in different lung pathologies.

S1PL is the terminal enzyme in S1P catabolism and hydrolyses S1P to ∆2-hexadecenal and ethanolamine phosphate. S1PL has been implicated in cancer [50], rheumatoid arthritis [51], sepsis [52] and pulmonary fibrosis [23]. S1PL is up-regulated in ovarian cancer [53], skin of atopic dermatitis patients [54], lung tissues from idiopathic pulmonary fibrosis patients [23], lungs from bleomycin instilled mice [23], and in lung tissues and BAL fluids from mice subjected to MV (Figure 1). The increased S1PL expression in LPS- [34] and VILI-induced mouse lungs (Figure 1) correlated with decreased S1P levels; however, in pulmonary fibrosis both SphK1 and S1PL expressions were elevated and blocking S1PL accentuated bleomycin-induced pulmonary fibrosis [23] suggested a protective role of S1PL in fibrosis.

The potential significance of these in vivo findings on protection of lung injury and apoptosis by S1PL were confirmed in vitro by subjecting alveolar epithelial cells to cyclic stretch (CS). CS regulates several cellular processes including cell adhesion [55], cell migration [56], oxidative stress [57], induction of inflammatory mediators [58,59,60] and signaling pathways that affect cell survival and apoptosis [61]. Cyclic stretch has been known to induce reorganization of the extracellular matrix by reorientation of the stress fibers. This leads to alteration in the cell-cell adhesions as well as the cell-extracellular matrix interactions. CS was also found to modulate the unfolding of inflammatory cascade in the cells [62]. Inhibiting S1PL with 4-DP, an analog of pyridoxal phosphate that is required for S1PL activity [32,63] attenuated CS induced gap formation and apoptosis in the alveolar epithelium. S1P plays an important role as a barrier stabilizing lipid mediator [64]. Subjecting the cells to pathophysiological levels of 18% CS was found to increase ceramide levels and reduce S1P levels, correlating with the in vivo data. CS was found to mediate apoptosis as seen by cleavage of caspase-3 and PARP which was reversed by inhibiting S1PL. Previous studies have shown that caspase mediated alveolar epithelial apoptosis is a major contributor to lung injury [65]. Caspase-3 is one of the executioner molecules which is activated as indicated by its cleavage to activated p17 and p12 fragments. Cleavage of PARP which is a downstream of Caspase-3 prevents the depletion of inadequate ATP storage in the cell leading to apoptosis of the cell [66]. This can be attributed to the fact that inhibiting S1PL will lead to restoration of S1P levels in the cells which have a pro-survival effect. S1P not only has a role in cell survival, but also plays a vital role in maintaining barrier function in the cells [16] and inhibition of S1PL in epithelial cells by 4-DP resulted in restoration of CS-induced paracellular gaps.

In summary, the current study demonstrates for the first time that mechanical stress in vivo and in vitro modulates sphingolipid levels and sphingolipid metabolizing enzymes that contribute to alveolar epithelial cell apoptosis, paracellular gap formation as well as cytokine release. During MV and CS, increased expression of S1PL and its activity enhanced degradation of S1P to ethanolamine phosphate and hexadecenal resulting in reduced S1P levels that contributed to epithelial cell apoptosis, barrier dysfunction and secretion of inflammatory cytokines. Therefore, inhibition of S1PL or activation of SphKs should offer protection and serve as a beneficial therapeutic strategy against VILI.

## 4. Materials and Methods

### 4.1. Materials

The mouse lung alveolar epithelial cell line, MLE12, was obtained from American Type Cell Culture (Manassas, VA, USA) and cultured in DMEM medium with 10% FBS and 1% Pen-Strep at 37 °C in 5% CO_2_. Phosphate-buffered saline (PBS) was obtained from Biofluids Inc. (Rockville, MD, USA). Pen-Strep, fetal bovine serum (FBS), trypsin, Triton X-100, Tween 20, phallaoidin and monoclonal β-Actin antibody were all obtained from Sigma-Aldrich Inc. (St. Louis, MO, USA). Cyclic stretch plates were purchased from Flexcell International (McKeesport, PA, USA). BCA protein assay kit was purchased from Pierce Chemical (Rockford, IL, USA). Novex-Tris Glycine gels were obtained from ThermoScientific (Waltham, MA, USA). The electro chemiluminescence kit was from Amersham Biosciences (Piscataway, NJ, USA). S1P Lyase antibody was purchased from Santa Cruz (Santa Cruz, CA, USA); SphK1 and SphK2 from Abcam (Cambridge, MA, USA); PARP, Caspase-3 and E-Cadherin from Cell Signaling (Danvers, MA, USA). 4-deoxypyridoxine (hydrochloride) was obtained from Cayman Chemical (Ann Arbor, MI, USA). ELISA kits were obtained from Peprotech (Rocky Hill, NJ, USA). FITC Annexin V Apoptosis Detection Kit was obtained from BD Biosciences (San Jose, CA, USA).

### 4.2. Murine Model of VILI

C57BL/6J mice were mechanically ventilated at 0 PEEP (Positive End Expiratory Pressure), 30 mL/kg for 4 h, lungs were lavaged, lipids from lung tissues were extracted [67] and analyzed by LC-MS/MS to quantify the sphingolipid levels [68]. *Sgpl1* heterozygous mice (*Sgpl1*^+/−^) were provided by Philip Soriano (Seattle) and *Sgpl1^+/+^* were derived by breeding the heterozygous mice [33]. *Sphk1^−/−^* mice were obtained from Richard L. Proia (NIH, Bethesda) [69]. All animal experiments were conducted in accordance with the procedures approved by the Institutional Animal Care and Use committee at UIC (ACC# 12-246, 21 February 2013). These animals were placed on mechanical ventilator (Harvard Apparatus, Boston, MA) for 4 h with high tidal volume ventilation (30 mL/kg, 75 breaths per minute and 0 PEEP, HTV), as mentioned in literature [70]. Control animals were anesthetized and allowed to breathe spontaneously. After 4 hours, bronchoalveolar lavage (BAL) was performed with an intratracheal injection of 1 mL of PBS solution followed by gentle aspiration. The recovered BALF was processed for protein concentration, total and differential cell counts, cytokine levels measured by ELISA. Lungs from the mice were collected for histologic evaluation by hematoxylin & eosin and TUNEL staining. Lung injury was scored based on the official workshop report of the American Thoracic society to distinguish the features during acute lung injury [71]. The expression of sphingolipid metabolizing enzymes was identified in the lung tissue lysates using western blotting. Sphingolipid levels were measured by liquid chromatography–tandem mass spectrometry (LC-MS/MS).

### 4.3. Analyses of Sphingoid Base-1-Phosphates

Analyses of sphingoid base-1-phosphates was performed by electrospray ionization liquid chromatography–tandem mass spectrometry, as previously described. The instrumentation used was an API4000 Q-trap hybrid triple-quadruple linear ion-trap mass spectrometer (Applied Biosystems, Foster City, CA, USA), equipped with a turbo ion spray ionization source interfaced with an automated Agilent 1100 series liquid chromatograph and auto sampler (Agilent Technologies, Wilmington, DE, USA). S1P and DihydroS1P were analyzed as bis-acetylated derivatives, with C17-S1P as the internal standard using reverse-phase high-performance liquid chromatography separation, negative-ion electrospray ionization, and magnetic resonance mammography analysis [68]. 

### 4.4. Cell Culture and Cyclic Stretch Experiments

MLE-12 were seeded onto six-well BioFlex plates coated with type I collagen (Flexcell), grown to confluence, and then exposed to pathological and physiological (18% and 5% elongation respectively) CS for 48 h, whereas the static plate was placed in the same incubator as the control. After 48 h, the lipids were extracted from the cells and the sphingolipid levels were measured by liquid chromatography–tandem mass spectrometry (LC-MS/MS). The expression of sphingolipid metabolizing enzymes and apoptotic markers were identified in the cell lysates using western blotting. Prior to subjecting the cells to CS, they were pretreated with 4-DP to inhibit S1P Lyase activity in the cells. Flow cytometry was used to identify the late apoptotic cells using FITC-Annexin V and PI staining. CS- induced gap formation between the cells was studied by staining the cells using F-actin and E-Cadherin. The secretion of cytokines was measured from the supernatants using ELISA. Densitometry analysis and gap formation computation was performed using ImageJ software (https://imagej.nih.gov/ij/). The date here was part of a work of the thesis of Vidyani Suryadevara for her Masters in Science [72]. Detailed methods have been stated in the Supplementary methods section of this manuscript.

### 4.5. Statistical Analysis

Experimental results are expressed as means ± SD of triplicate values from three independent experiments. All results were subjected to statistical analysis using one-way ANOVA and, whenever appropriate, analyzed by Student-Newman-Keul’s test *p* < 0.05 was considered statistically significant.

## 5. Conclusions

Sphingolipids have been found to play a very important role in various lung diseases [16,25,26,27,34,40,41,47]. In this study, the role of sphingolipids and sphingolipid metabolizing enzymes in VILI was identified. VILI leads to distention and damage of the alveolar epithelium, release of cytokines that mediate systemic inflammation and several other mechanisms mediating lung injury [4]. Ceramide levels were found to be upregulated in lung tissues of mice subjected to mechanical ventilation, in addition to the reduction in sphingosine-1-phosphate levels (Figure 1). These results are concurrent with the already established facts that ceramide mediates apoptosis [14], while S1P has a pro-survival effect [16]. Among the sphingolipid metabolizing enzymes, S1P Lyase protein expression was elevated in lung tissues and BALF of mice subjected to VILI. This enhanced S1P Lyase led to reduction in S1P levels. We observed that *Sgpl1^+/−^* mice, wherein S1P levels were restored, were protective against VILI than wildtype mice (Figure 2). As a proof of principle, it was found that VILI was accentuated in *SphK1^−/−^* mice which have reduced S1P levels (Figure 3). Thus, these in vivo studies have elucidated the vital role of the balance of S1P levels in the lung in mitigating ventilation-mediated lung injury.

Alveolar epithelial apoptosis was prevalent in mice subjected to ventilation and *Sgpl1^+/-^* mice were found to have less apoptosis cells upon MV than wildtype mice (Figure 4). Further, it was observed that alveolar epithelial cells subjected to pathophysiological levels of cyclic stretch (18% CS) had an increase in ceramide levels, S1P Lyase protein expression and reduction in S1P levels in congruence with the in vivo data (Figure 5). Inhibition of S1P Lyase using 4-deoxypyridoxine, an analog of pyridoxal phosphate reduced 18% CS induced apoptosis (Figure 6), paracellular gap formation and release of cytokines like IL-6 and TNF-α (Figure 7). The findings from these studies elucidate that restoring S1P levels by inhibiting S1P Lyase mitigates VILI and thus further S1P Lyase inhibitors can be potential therapeutic targets against VILI.

## Supplementary Materials

Supplementary materials can be found at www.mdpi.com/1422-0067/19/1/114/s1.
