# Supplementary material for: Sphingolipids in Ventilator Induced Lung Injury: Role of Sphingosine-1-Phosphate Lyase
Source: Int J Mol Sci. 2018 Jan 1;19(1):114. doi: 10.3390/ijms19010114 (PMC5796063; doi:10.3390/ijms19010114)
Supplement: Supplementary file 1 [file ijms-19-00114-s001.pdf]

International Journal of
Molecular Sciences

MDPI

Supplementary materials and methods:

MLE2 cell culture and cyclic stretch: Mouse lung epithelial cells (MLE-12) were purchased from ATCC (Manassas, VA) and cultured in DMEM medium with 10% FBS and 5% pen-strep at 37°C in 5% CO_2 and seeded onto BioFlex plates. Plates were subjected to stretch to produce either 5% or 18% elongation at a frequency of 0.5 Hz, 30 cycles/min by controlled vacuum in the device were placed on a Flexcell Strain Tension System (FX-3000, Flexcell International, Hillsborough, NC) kept in a 5% CO_2 incubator at 37^0C and 95% humidity. Static plate was placed in the same incubator to retain similar conditions. As we have previously reported, 18% cyclic stretch (CS) corresponds to pathologically relevant levels of mechanical stress, whereas 5% cyclic stretch corresponds to physiological level of mechanical stress.

Mice: Heterozygous mice ($S1PL^{+/-}$) were provided by Dr. Philip Soriano (Seattle) and *Sphk1*$^{-/-}$ mice were obtained from Dr. Richard L. Proia (NIH, Bethesda, MD). The animals were bred and housed in a specific pathogen-free barrier facility maintained by the University of Illinois, Biologic Resources Laboratory. All experiments were conducted with protocols approved by the Institutional Animal Care and Use Committee of the University of Illinois at Chicago.

Pre-clinical model of VILI in mice: Subjecting normal mice to ventilation leads to ventilator induced lung injury and this animal model has been widely used to study VILI. Adult mice with an average weight of 20-25 grams were used for the experiments. The mice were anesthetized by intraperitoneal injection of ketamine (100mg/kg) and xylazine (5mg/kg). Tracheotomy was performed in mice, and the trachea was cannulated with a 20-gauge, one-inch catheter (Penn-Century, Philadelphia, PA) that was tied into place to prevent air leak. These animals were placed on mechanical ventilator (Harvard Apparatus, Boston, MA) for 4 hours with high tidal volume ventilation (30 ml/kg, 75 breaths per minute and 0 PEEP, HTV). Mice concurrently received intraperitoneal injection of sterile PBS solution (every 30-minute interval) during mechanical ventilation. Control animals were anesthetized and allowed to breathe spontaneously.

Assessment of lung inflammation and morphology: After the experiment, animals were sacrificed by exsanguination under anesthesia. Bronchoalveolar lavage (BAL) was performed using 1ml of sterile PBS and measurements of cell count and protein concentration were conducted. The BAL protein concentration was determined by a modified Lowry colorimetric assay using a Bio-Rad DC protein assay kit (BioRad Laboratories, Hercules, CA). BAL inflammatory cell counting was performed using a standard hemocytometer technique. Differential cell counts were performed after staining the cells with Diff-Quik stain kit (Cat# B4132- 1A, Dade Behring, DE, USA). Cytokine levels in cell-free BAL fluid samples and supernatants were measured using ELISA (Peprotech), and the values were expressed as picograms per milliliter of the BAL fluid.

Lung injury score: For histological assessment of lung injury, the left lung was excised from mice, and lobes were sectioned, embedded in paraffin, and cut into 5μm sections. Hematoxylin and eosin (H&E) was performed by the Pathology Core Facility (University of Illinois–Chicago). The representative images of the lung tissue sections (15 per lung section) were acquired with a DXM1200 digital camera (Nikon, Tokyo, Japan) at **20X** magnification. Lung injury score was assessed from the lung tissue sections by identifying specific histological features from multiple sections across the lung tissue. The score is calculated using the formula below by using five independent variables as indicated in the table below:

TABLE 1. LUNG INJURY SCORING SYSTEM

	Score per field		
Parameter	0	1	2
A. Neutrophils in the alveolar space	none	1–5	>5
B. Neutrophils in the interstitial space	none	1–5	>5
C. Hyaline membranes	none	1	>1
D. Proteinaceous debris filling the airspaces	none	1	>1
E. Alveolar septal thickening	<2x	2x–4x	>4x

Score = [(20 × A) + (14 × B) + (7 × C) + (7 × D) + (2 × E)]/(number of fields × 100)

The right lung from each mouse was collected and homogenized, these tissue lysates were prepared for further analysis.

Analysis of Sphingoid Base-1-Phosphates: Analyses of sphingoid base-1-phosphates was performed by electrospray ionization liquid chromatography–tandem mass spectrometry, as previously described. The instrumentation used was an API4000 Q-trap hybrid triple-quadruple linear ion-trap mass spectrometer (Applied Biosystems, Foster City, CA), equipped with a turbo ion spray ionization source interfaced with an automated Agilent 1100 series liquid chromatograph and autosampler (Agilent Technologies, Wilmington, DE). S1P and DihydroS1P were analyzed as bis-acetylated derivatives, with C17-S1P as the internal standard using reverse-phase high-performance liquid chromatography separation, negative-ion electrospray ionization, and magnetic resonance mammography analysis.

TUNEL Assay: End labeling of exposed 3′-OH ends of DNA fragments was undertaken with the TUNEL *in situ* cell death detection kit Alkaline Phosphatase (Roche Diagnostics, Indianapolis, IN), as per the manufacturer's instructions. The TUNEL index was calculated by counting the total number of TUNEL+ cells. At least two sections were used from each mouse for analyses.

Cyclic stretch-induced apoptosis of epithelial cells by flow cytometry: the apoptosis of MLE12 cells subjected to cyclic was assayed by flow cytometry with the use of FITC Annexin V Apoptosis Detection Kit (BD Biosciences, San Diego, CA). The annexin V apoptosis assay was performed according to the manufacturer's recommendations. The cells subjected to cyclic stretch as well as static cells were suspended using Cellstripper (non-enzymatic cell dissociation solution) and collected using centrifugation. The cells were suspended in 500 mL of 1X binding buffer, and 5 mL of annexin V-FITC and propidium iodide (PI) were added according to the manufacturer's instructions (Biovision, Inc., Mountain View, CA, USA). After incubation at room temperature for 5 min in the dark, the cells were evaluated for annexin V-FITC and PI binding by flow cytometry using a FACSCalibur (BD Biosciences, Inc., San Jose, CA, USA). Apoptotic cells were identified by double labelling with Annexin V and PI or by labelling with only annexin V. PI labels all dead cells, including necrotic cells and cells in late stages of apoptosis; cells entering early apoptosis are stained only by annexin V, and the viable cells do not stain with annexin V or PI.

Further, apoptosis was analyzed by western blotting of apoptosis markers Caspase-3 and PARP. **Caspases** are the most critical molecules for the execution of apoptosis, out of which Caspase-3, once activated executes apoptosis by cleaving a variety of substrates, or "death substrates". Activation of caspase-3 results in the cleavage of the 34KDa into activated 17KDa and 12KDa fragments. PARP, a 116 kDa nuclear poly (ADP-ribose) polymerase, is a substrate for Caspase -3. It is a DNA repair enzyme, which when cleaved loses its activity and alleviates cellular disassembly. During the cleavage of PARP, the carboxy-terminal catalytic domain (~89 kDa) is separated from the amino-terminal DNA binding domain (~24 kDa). In this study, the cell lysates from MLE-12 cells subjected to different degrees of cyclic stretch were analyzed and

detection of ~89 kDa PARP fragment with anti-PARP thus serves as an early marker of apoptosis. Cell lysates from MLE-12 cells subjected to cyclic stretch were probed for western blotting using anti-PARP and anti-Caspase antibody. The cleavage of Caspase-3 and PARP after cyclic stretch indicates apoptosis. The extent of apoptosis was further confirmed by sorting the cells as viable cells, early apoptotic cells, late apoptotic and nectrotic cells.

Immunofluorescence Microscopy: MLE-12 cells were subjected to cyclic stretch upon pretreatment with 4DP (1mM) for 90 min. Cells were fixed in 3.7% para-formaldehyde in PBS for 10 min, washed three times with PBS, permeabilized for 4 min in 3.7% paraformaldehyde containing 0.25% Triton X-100, blocked with 2% BSA in TBST, incubated for 1 h with appropriate primary antibody (1:200 dilution), washed with TBST, and stained for 1 h with secondary antibody (1:200 dilution) in TBST containing 2% BSA. Cells were examined using a Nikon Eclipse TE2000-S immunofluorescence microscope and a Hamamatsu digital camera with ×60 oil immersion objective and Meta Vue software (Universal Imaging Corp., PA, USA). E-Cadherin and actin dynamics was also investigated using Zeiss 510 Meta laser scanning microscope. Quantification of paracellular gap formation was performed as described earlier. Briefly, for each image, background signal was subtracted by drawing a region of interest around the cell periphery of individual cells. All areas outside the cell were cleared to best visualize the leading edges including cell periphery and the fluorescence intensity within the entire cell was quantified by MBF ImageJ bundle.
